# Second Diagnostic Opinion by Experienced Dermatopathologists in the Setting of a Referral Regional Melanoma Unit Significantly Improves the Clinical Management of Patients With Cutaneous Melanoma

**DOI:** 10.3389/fmed.2020.568946

**Published:** 2021-02-04

**Authors:** Andrea Ronchi, Francesca Pagliuca, Federica Zito Marino, Giuseppe Argenziano, Gabriella Brancaccio, Roberto Alfano, Giuseppe Signoriello, Elvira Moscarella, Renato Franco

**Affiliations:** ^1^Pathology Unit, Department of Mental and Physical Health and Preventive Medicine, University of Campania “Luigi Vanvitelli,” Naples, Italy; ^2^Dermatology Unit, Department of Mental and Physical Health and Preventive Medicine, University of Campania “Luigi Vanvitelli,” Naples, Italy; ^3^Department of Anaesthesiology, Surgery and Emergency, University of Campania “Luigi Vanvitelli,” Naples, Italy; ^4^Department of Mental and Physical Health and Preventive Medicine, University of Campania “Luigi Vanvitelli,” Naples, Italy

**Keywords:** melanoma, second diagnostic opinion, diagnostic agreement, clinical management, caseload, melanocytic neoplasms

## Abstract

The diagnosis of cutaneous melanoma and melanocytic neoplasms in general is one of the most challenging fields in pathology, and the reported interobserver diagnostic agreement in the evaluation of melanocytic lesions is poor. Nevertheless, a correct histopathological diagnosis is crucial to ensure a good clinical management of the patients. The institution of multidisciplinary teams has recently modified the approach to the patients with cutaneous melanoma. Patients referred to a multidisciplinary melanoma unit after receiving a diagnosis of melanoma elsewhere are encouraged to have their histopathological diagnosis confirmed by a second opinion from the experienced pathologist of the team before any treatment is initiated. We performed a retrospective analysis on a series of 121 histopathological revisions required for melanocytic neoplasms in the context of a multidisciplinary team, in order to evaluate the effects of second diagnostic opinion (SDO) on the clinical management of the patients. We defined three types of diagnostic discrepancies between the first diagnosis and the second opinion, according to the greatness of their clinical impact. Overall, the incidence of diagnostic discrepancies of any type was quite high in our series (56%). Interestingly, the SDO determined relevant changes in the clinical management of the patients in 33 out of 121 (27.3%) cases. This study confirms that SDO by expert pathologists significantly affects the course of treatment of melanoma patients and helps improving the diagnostic accuracy and clinical outcome.

## Introduction

Cutaneous melanoma (CM) is an aggressive tumor, with a 5-year survival rate of only 15–20% in advanced stage (1). Despite the latest improvements in prevention strategies and public attention focusing on the need of reducing UV exposure, the incidence of CM is increasing worldwide (2). Nowadays, CM is a significant cause of public health expenditure, as the annual cost for melanoma patients' treatment in the United States is expected to reach almost $1.6 billion in 2030 (2). Simultaneously, as the Western World population is gaining awareness of CM as a public health issue, an increasing number of patients are attending clinical and dermoscopical screening tests. The recent development and large-scale availability of dermoscopy have greatly enhanced the ability to early recognize those melanocytic lesions requiring excision (3). Consequently, as the diagnosis of CM is currently based on the pathological examination, the pathologists are often called to diagnose CM in an early stage (4). The pathological diagnosis of CM is tricky for several factors. Firstly, despite the recent findings about the molecular and genetic features of melanocytic neoplasms, the pathological diagnosis of CM still mainly relies on “basic” morphology (5–7). Among the listed morphologic criteria, most are qualitative rather than quantitative and their evaluation is at least partially subjective. In addition, although the diagnosis of a CM with robust morphological features in an adult patient can be relatively easy, rarer histotypes, and cases of CM in young patients might result difficult to be interpreted. Finally, CM has a wide spectrum of histological mimickers and several benign and borderline melanocytic lesions with overlapping morphological features have to be considered in the differential diagnosis. In this context, improvements have been reached in the last years in immunohistochemistry and molecular tests, particularly in some specific diagnostic settings, including BAPomas, nevoid melanomas, and atypical spitzoid lesions. Nevertheless, the diagnosis of melanocytic lesions is still strongly based on morphological findings in many cases (8, 9). Moreover, comprehensive pathological evaluation not only must distinguish between a nevus and a CM but also should correctly assess all these features to allow a proper staging and, thus, clinical management of the patients (10). The diagnosis of melanocytic lesions is currently one of the biggest challenges for pathologists, and a general pathologist may not have enough experience to express a correct diagnostic orientation, mainly when facing less conventional cases (11). Therefore, a second diagnostic opinion (SDO) from a pathologist with a high expertise in dermatopathology could improve the diagnostic accuracy in cases of melanocytic neoplasms firstly diagnosed by a general pathologist (11). In this context, the institution of multidisciplinary oncological teams brought about a revolution in CM patient management, and the convergence of dermatological and pathological skills allows to improve the accuracy of the diagnosis (11). The institution of Melanoma Unit Multidisciplinary Group, including dermatologists, pathologists, oncologists, surgeons, radiologists, radiotherapists, and molecular biologists, is considered an important step toward the improvement of the standard of care. Thus, all patients admitted to such Unit are treated with a multidisciplinary approach. In our Unit, the Melanoma Unit has a large caseload of melanocytic lesions, with more than 400 diagnosed CMs per year. Most patients receive their first diagnosis of CM directly within the Melanoma Unit while a small but still considerable number of patients are admitted to the Melanoma Unit only after receiving a diagnosis of melanocytic lesion elsewhere. Particularly, territorial dermatologists address their patients to the Melanoma Unit, either when a diagnosis of melanoma is provided or when the suspicion of melanoma persists despite that the histological diagnosis is not made. In such cases, the original histological biomaterials are required to obtain a SDO by the expert pathologists of the Unit.

In the present work, we performed a retrospective analysis on the melanocytic lesions referred to the Melanoma Unit of University “Luigi Vanvitelli” (Naples, Italy) for SDO, all addressed and filtered on a clinical viewpoint, with the aim of evaluating the effect of SDO by experienced dermatopathologists on the clinical management of patients with diagnosis of CM in a referral regional interdisciplinary group.

## Materials and Methods

### Second Diagnostic Opinion

Original slides and paraffin-embedded tissue blocks from 130 melanocytic lesions were received for SDO by the referral pathologists of the Melanoma Unit of University “Luigi Vanvitelli” (Naples, Italy), between November 2018 and February 2020. All the cases had been previously diagnosed by general pathologists in peripheral Hospitals and private diagnostic centers and, more rarely, by expert pathologists of other Institutions with a large caseload of melanocytic lesions. All these consecutive 130 cases were considered for the present study. The only exclusion criterion was the existence of technical problems affecting the quality of the received biological materials, which prevented a satisfactory reevaluation. Therefore, nine cases were not included in this series, as they were considered non-diagnostic due to technical reasons. All the remaining 121 cases included in the present study had been reevaluated by two experienced dermatopathologists of the Melanoma Unit to reach a SDO. In all the cases, clinical information and the first pathological diagnosis were obtained from the original diagnostic reports. The original histological slides were reexamined, and additional hematoxylin-and-eosin-stained slides (two by default) were cut from the received tissue blocks. After independent evaluation of the original and newly obtained histological sections, the two referral pathologists shared their opinions on the cases and multiple deeper levels, immunohistochemical stains, and/or molecular tests were ordered when considered useful. Ancillary studies were considered mandatory especially when there was no consensus between the two referral pathologists on the diagnosis or when their diagnostic orientation significantly differ from the first diagnosis. The cases were then further discussed until a diagnostic agreement was reached. Routinely ordered antibodies were p16, HMB-45, and Ki-67. For immunohistochemistry, the Nuclear Fast Red Counterstain protocol was preferred when evaluating hyperpigmented lesions. Other immunostainings which were more or less frequently ordered included S100, Melan-A, SOX10, cytokeratins. When morphological features suggesting the possibility of ALK- or NTRK-fused or BAP1-mutated melanocytic lesions were observed, the cases were immunohistochemically stained for ALK, TRK, or BAP1 (12–14). Fluorescent *in situ* hybridization (FISH) analysis was performed in the most challenging cases. The FISH panel which was used consisted of the following probes: RREB1, MYB, CEN6, CCND1, and CDKN2A. For the present study, the first diagnoses were compared with the second diagnoses, paying attention to both the final diagnostic formulations and the assessment of the pathological parameters influencing prognosis, therapy, and clinical management of the patients. Furthermore, we defined as “center with a large caseload” a center with more than 100 CMs diagnosed in a year, and as “center with small caseload” a center with <100 CMs diagnosed in a year. Moreover, we calculated the time interval between the first diagnoses and the SDOs.

### Diagnostic Discrepancies

We defined three types of diagnostic discrepancies, to facilitate comparison and discussion of the results:

Type I: discordant diagnostic category (i.e., first diagnosis of benign nevus or nevus of uncertain malignant potential, second diagnosis of malignant melanoma, and *vice versa*).
Type II: concordant diagnostic category with secondary major discrepancies, affecting therapy and clinical management of the patient (i.e., first staging pT1a and second staging pT1b).
Type III: minor discrepancies, with prognostic relevance, but not affecting the therapy and the clinical management of the patient (i.e., different Breslow thickness, but in the same staging range).

### Statistical Analysis

Statistical analysis was carried out using IBM SPSS statistics 20. The categorical variables for both type I and type II discrepancies, highly impacting on patients' management, were tabulated, and the agreement rate between the external and the referral pathologists was calculated. The Cohen's kappa coefficient (κ) of agreement was estimated as a measure of the agreement (15). Kappa values from 0 to 0.2, 0.21 to 0.4, 0.41 to 0.6, 0.61 to 0.8, and 0.81 to 1.0 indicate none, minimal, weak, moderate, strong agreement, respectively. In addition, the Cohen's kappa coefficient was also estimated considering the external pathologist as working in a small or a large caseload center. The MCNemar's test was used to compare the discrepancies between the two observers. In order to evaluate the significant efficacy of the SDO on the change of patients' management in our series, using Person's χ^2^ we considered the presence of consistent revision (cases with types I and II discrepancies) and inconsistent revision (no discrepancy and type III discrepancies), establishing their relations with the change of treatment, i.e., re-biopsy only, re-biopsy plus sentinel node biopsy, or sentinel node biopsy only. This analysis was also conducted considering also small and large caseload sub-groups. Differences were significant for values of *p* < 0.05.

## Results

### Clinical Features

Our Series of 121 patients included 59 (48.8%) males and 62 (51.2%) females, with a median age of 50.6 years (ranging from 15 to 86). The lesions were located on the back in 40 (33.1%) cases, on the trunk in 31 (25.6%) cases, on the upper limbs in 12 (9.9%) cases, on the lower limbs in 27 (22.3%) cases, and in the head-and-neck region in 11 (9.1%) cases. All the lesions were excised because considered clinically and dermoscopically suspicious for melanoma. Overall, 95 out of 121 cases (78.5%) came from centers with a small caseload, while the remaining 26 cases (21.5%) came from centers with a large caseload.

### Histopathological Diagnoses Before and After the SDO

**Figure 1:**
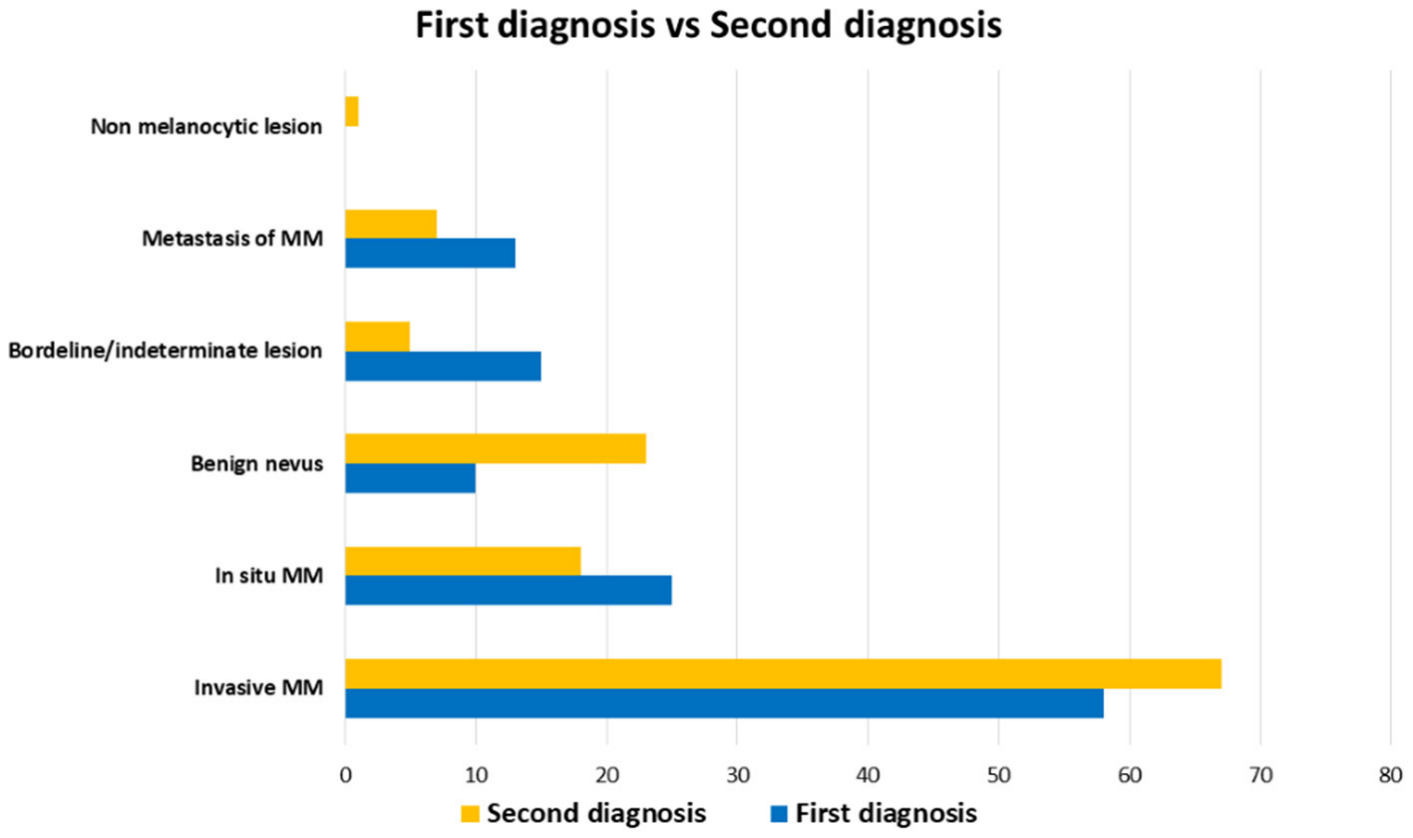
Distribution of diagnoses in the Series. First diagnoses (blue) and second diagnostic opinions (yellow) are compared. Benign nevi and invasive malignant melanoma were diagnosed more frequently by second diagnostic opinion than first diagnoses, while borderline/indeterminate lesions are more frequent in first diagnoses.

**Figure 2:**
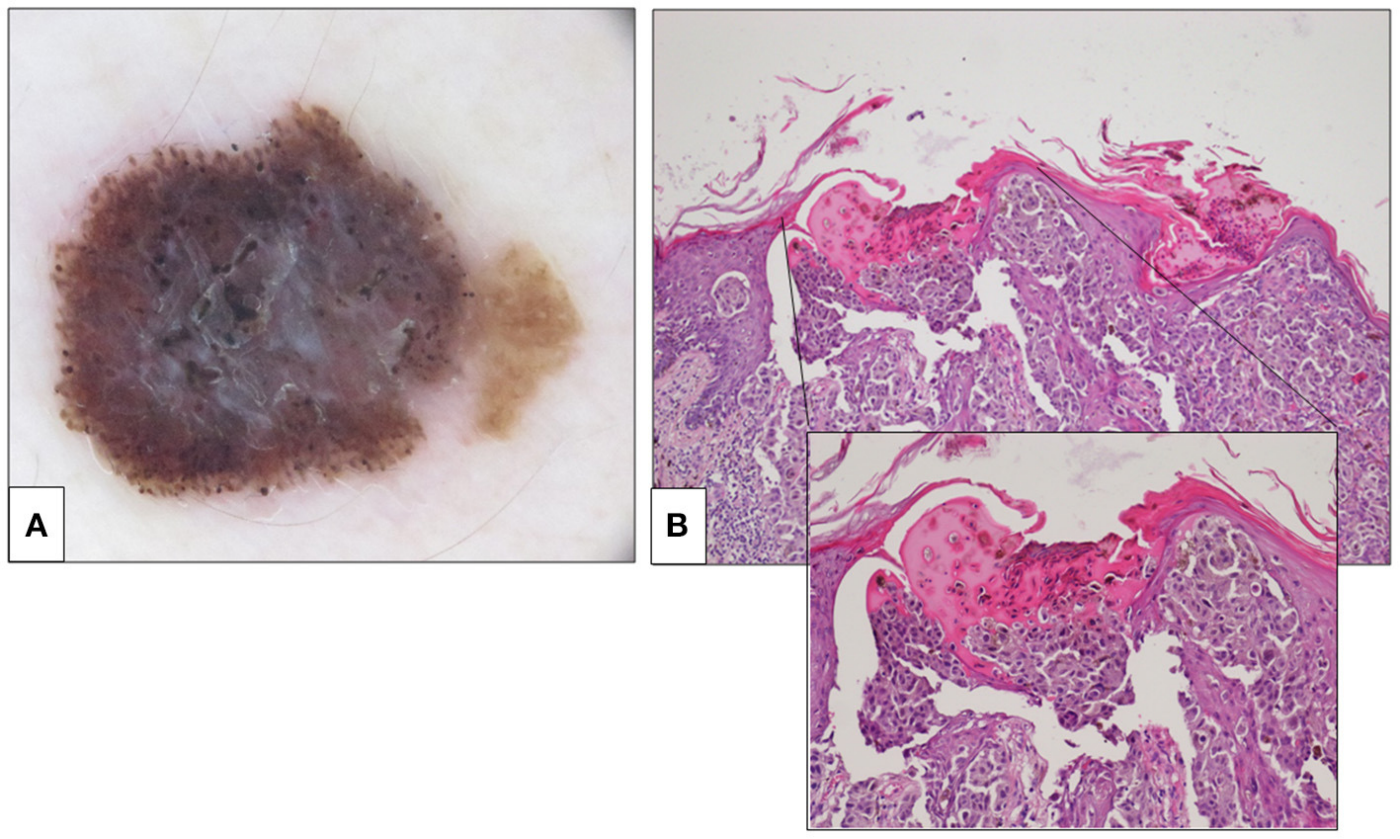
Fifty five-year-old woman, right leg. **(A)** Dermoscopic features. An asymmetric pigmented lesion displaying irregular globules at the periphery. In the central area, multiple small micro-erosions are visible in the context of a blue-white veil. **(B)** Histological features showed a 1.3-millimeter Breslow thickness melanoma (H&E, 10×). Multiple levels were examined to demonstrate the microscopic ulceration (inset, H&E 40×). The histological demonstration of the microscopic ulceration led to a change of the stage (from pT2a to pT2b). H&E, hematoxylin and eosin.

**Figure 3:**
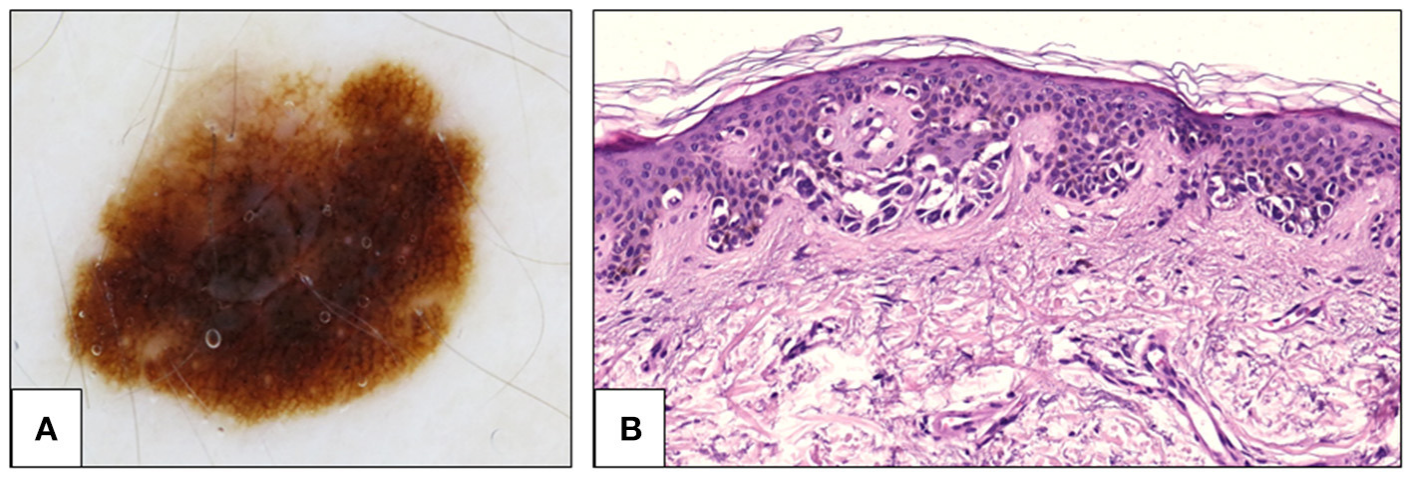
Fifty two-year-old woman, back. **(A)** Dermoscopy showing atypical pigment network. The network is prominent, with thin lines and abrupt ending at the periphery. Histological examination showed an asymmetrical proliferation of melanocytes that appeared organized in large, confluent, irregularly shaped nests and in a continuous lentiginous pattern. **(B)** At the periphery of the lesion, pagetoid spread of single atypical melanocytes was seen (H&E, 20×). The diagnosis was changed from SAMPUS to *in situ* melanoma. H&E, hematoxylin and eosin; SAMPUS, superficial atypical melanocytic proliferations of unknown significance.

**Figure 4:**
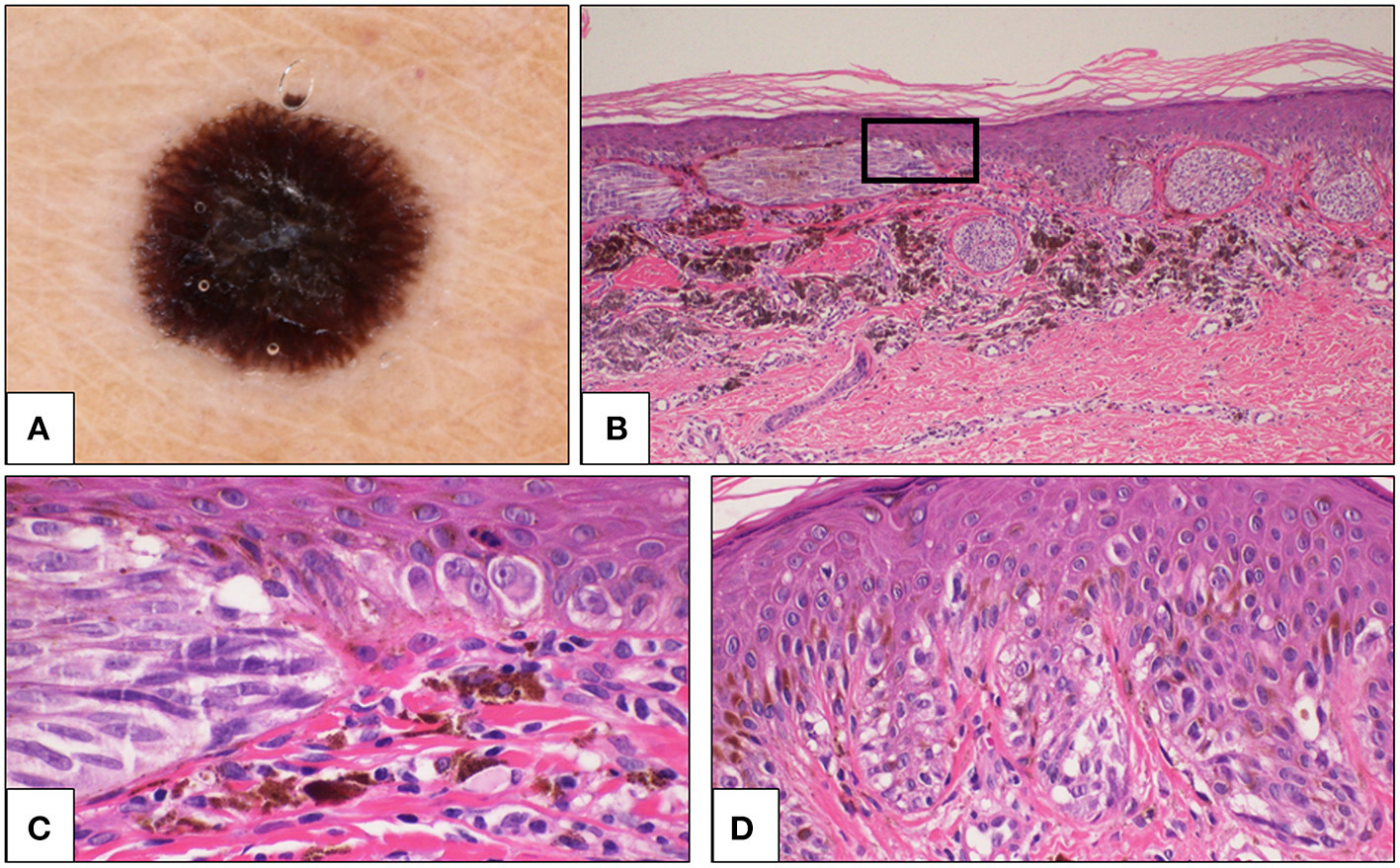
Sixty one-year-old woman, thoracic region. **(A)** Dermoscopy showing peripheral streaks and black blotch in the center of the lesion. **(B)** Histologically, large junctional nests were a prominent feature, and some nests were parallel to the epidermis. A large amount of melanophages was present in the dermis below the lesion (H&E, 4×). The rectangular area is magnified in **C**. **(C)** Cells are large, spindle-shaped in the nest (left side) and epithelioid in the lentiginous component of the lesion (right side) (H&E, 40×). **(D)** In some fields, a continuous lentiginous pattern of growth and some pagetoid elements were evident (H&E, 20×). The diagnosis was changed from Spitz nevus to malignant melanoma with spitzoid features. H&E, hematoxylin and eosin.

The diagnostic agreement between the two referral pathologists resulted high (115 out of 121 cases, about 95%). In the remaining 6 cases, a diagnostic agreement has been reached after a discussion between the two pathologists. On the basis of the first diagnoses, the Series included 58 (38%) invasive CMs, 25 (19.8%) *in situ* CMs, 10 (12.5%) benign nevi, 13 (4.2%) metastatic CMs, and 15 (15.3%) borderline/indeterminate melanocytic lesions including 7 (8.3%) SAMPUSes (superficial atypical melanocytic proliferations of unknown significance), 3 (2.8%) MELTUMPs (melanocytic tumors of uncertain malignant potential), and 5 (4.2%) ASTs (atypical Spitz tumor). After the SDO, the Series included 67 (55.4%) invasive MMs, 18 (14.9%) *in situ* melanomas, 23 (19%) benign melanocytic lesions, 5 (4.1%) borderline/indeterminate melanocytic lesions (4 cases of AST and one pigmented epithelioid melanocytoma), 7 (5.8%) metastatic MMs and 1 (0.8%) non-melanocytic lesion (dermatofibroma) (Figure 1). Figures 2–4 show the dermoscopic and histological features of some cases.

### Diagnostic Discrepancies and Diagnostic Agreement

**Figure 5:**
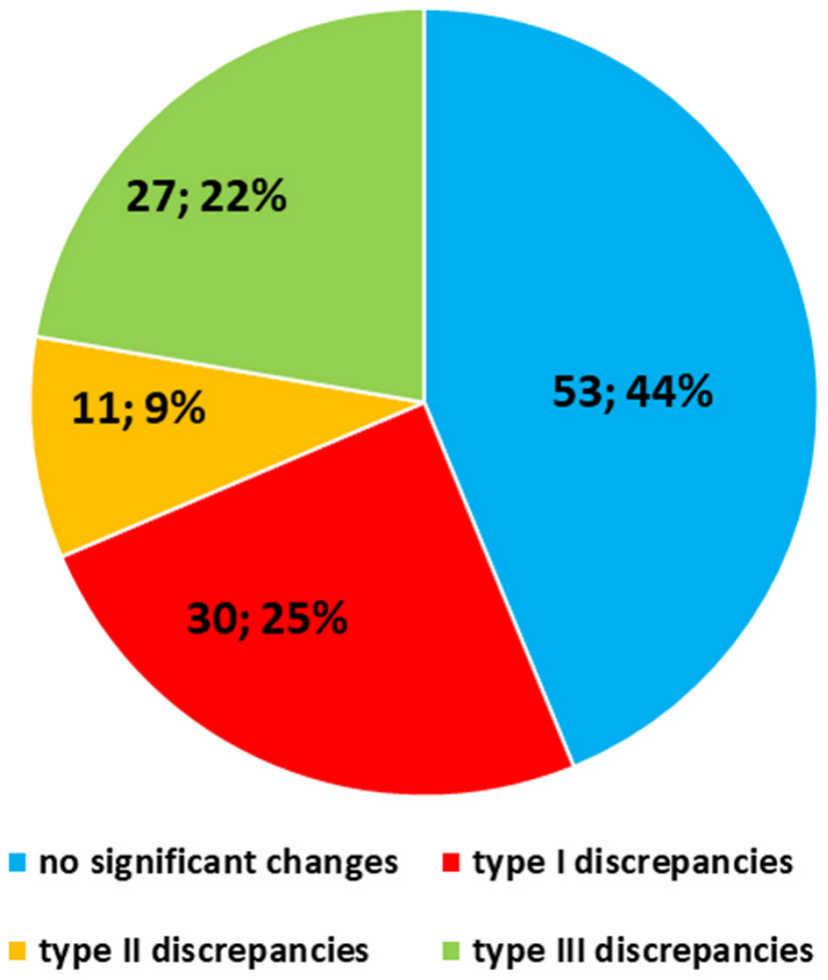
Quantitative distribution of diagnostic discrepancies in the Series. Overall, diagnostic discrepancies constituted 56% of the cases. In particular, type I discrepancies (discordant diagnostic category) allowed for 25% of all cases; type II discrepancies (concordant diagnostic category with secondary major discrepancies, affecting therapy and clinical management of the patient) allowed for 9%; type III discrepancies (minor discrepancies, with prognostic relevance, but not affecting the therapy and the clinical management of the patient) allowed for 22%.

The SDO did not change the diagnosis in 53 out of 121 (44%) cases, while diagnostic discrepancies were observed in the remaining 68 (56%) cases. Particularly, Type I discrepancies were observed in 30 out of 121 (25%) cases and Type II discrepancies were found in 11 out of 121 (9%) cases. Finally, Type III discrepancies were observed in 27 out of 121 (22%) cases (Figure 5). Thirty-five out of the 53 cases (66%) with no significant diagnostic discrepancy came from centers with a small caseload.

#### Type I Discrepancies

Type I discrepancies were found in 30 out of 121 (25%) of the cases. Particularly, Type I discrepancies included 10 out of 121 (8.3%) cases with the first diagnosis of melanoma (7 *in situ* CMs and 3 invasive CMs) and the SDO of benign nevus; 13 out of 121 (10.7%) cases with first diagnosis of undetermined malignant potential (SAMPUS, MELTUMP, AST) while the SDO was CM or benign nevus in 5 cases and in 8 cases, respectively, and in 5 out of 121 (4.1%) cases, the first diagnosis was benign nevus and the SDO was CM. In 1 out of 121 (0.8%) cases, the first diagnosis was Reed nevus and SDO was pigmented epithelioid melanocytoma. Finally, in 1 out of 121 (0.8%) cases the first diagnosis was desmoplastic Spitz nevus and the second opinion was dermatofibroma. Consequently, over-interpretation was observed in 15 out of 30 (50%) of the cases and under-interpretation in 14 out of 30 (46.7%) of the cases. In the remaining case, both the first diagnosis and the SDO diagnosed a benign lesion, even if the diagnostic category was different (desmoplastic Spitz nevus vs. dermatofibroma). Type I discrepancies are detailed in Table 1.

**Table 1:** Cases with type I discrepancies.

**Sex**	**Age**	**Site**	**Center caseload—first diagnosis**	**Ancillary tests for first diagnosis**	**First diagnosis**	**Second opinion**	**Ancillary tests for second opinion**
F	15	Shoulder	Small	–	RN	PEM	IHC
M	16	Right thigh	Small	–	AST	SN	IHC + FISH
F	18	Face	Small	IHC	SAMPUS	DN	IHC
M	19	Hip	Large	nr	MELTUMP	DN	IHC
F	22	Right leg	Small	nr	SN desmoplastic	Dermatofibroma	IHC
M	23	Back	Small	–	IM	CN	IHC
F	24	Back	Small	nr	ISM	DN	IHC
F	24	Back	Small	nr	ISM	DN	IHC
F	25	Back	Small	–	SAMPUS	DN with spitzoid features	IHC
F	26	Right thigh	Small	–	IM	DN with spitzoid features	IHC
M	27	Thorax	Small	nr	SAMPUS	ISM	IHC
M	28	Thorax	Small	IHC	ISM on nevus	DN	IHC
F	29	Right leg	Small	nr	IM with spitzoid features	SN	IHC + FISH
F	39	Back	Large	nr	SAMPUS	DN	IHC
M	39	Abdomen	Small	nr	ISM	DN	IHC
M	43	Back	Small	IHC	MELTUMP	IM	IHC
F	43	Neck	Small	nr	AST	IM	IHC
F	44	Back	Small	–	ISM	DN	IHC
M	47	Leg	Small	IHC	SAMPUS	IM	IHC
F	47	Buttock	Small	nr	ISM	DN	IHC
M	49	Back	Small	nr	CN	NM	IHC
F	52	Back	Large	IHC	SAMPUS	IM	IHC
M	53	Shoulder	Small	IHC	ISM	DN	IHC
M	59	Back	Small	IHC	DN	ISM	IHC
M	61	Back	Small	IHC	SAMPUS	ISM	IHC
F	61	Thorax	Small	nr	SN	ISM	IHC
F	62	Left arm	Small	–	MELTUMP	ISM	IHC
M	64	Face	Small	IHC	CN	ISM	IHC
M	70	Back	Small	IHC	AST	IM with spitzoid features	IHC
M	75	Thorax	Small	–	CN	ISM on nevus	IHC

*AST, atypical Spitz nevus; CN, common nevus; DN, dysplastic nevus; IM, invasive melanoma; ISM, in situ melanoma; MELTUMP, melanocytic tumor of uncertain malignant potential; NM, nodular melanoma; PEM, pigmented epithelioid melanocytoma; RN, reed nevus; SAMPUS, superficial atypical melanocytic proliferation of uncertain significance; SN, Spitz nevus; FISH, fluorescent in situ hybridization; IHC, immunohistochemistry; nr, not reported*.

Overall agreement between the external and referral pathologists in melanoma diagnosis resulted weak (*k* = 0.48, 95% CI, 0.30–0.66, *p* = 0.000). Considering the origin of the first diagnosis, type I discrepancies between the small caseload interpretation and the SDO interpretation were observed in 27 out of 30 (90%) cases, with over-interpretation in 15 (55.6%) cases and under-interpretation in 11 (40.7%) cases. The agreement between the external small caseload pathologist and the referral pathologist in melanoma diagnosis resulted minimal (*k* = 0.368, 95% CI, 0.16–0.56, *p* = 0.000). Type I discrepancies between the large caseload interpretation and the SDO interpretation were observed in 3 out of 30 (10%) cases, consisting in three cases of under-interpretation. The agreement between the external large caseload pathologist and the referral pathologist in melanoma diagnosis resulted strong (*k* = 0.859, 95% CI, 0.74–0.98, *p* = 0.000).

#### Type II Discrepancies

Type II discrepancies were found in 11 out of 121 (9%) cases, resulting in 6 cases of over-staging and 5 cases of under-staging. These discrepancies included different Breslow thickness in 9 out of 121 cases (7.4%) and the different assessment of ulceration in 1 out of 121 (0.8%) cases, determining a significant change in the staging of the tumor with clinical and therapeutic consequences. Differences in both Breslow thickness and ulceration were observed in the remaining 1 out of 121 (0.8%) cases. All the 11 Type II discrepancy cases came from centers with a small caseload. Diagnostic agreement between the external and the referral pathologists resulted strong (*k* = 0.89, 95% CI, 0.69–1.00, *p* = 0.000).

#### Type III Discrepancies

Twenty-seven of 121 (22%) cases presented Type III discrepancies. Particularly, 18 out of 121 (14.9%) cases showed a different Breslow thickness in the same staging range, 1 out of 121 (0.8%) cases showed discrepancies in evaluation of ulceration, and 2 out of 121 (1.6%) cases presented differences in both Breslow thickness and ulceration. In 6 out of 121 (5%) cases, only minor prognostic factors (number of mitoses, presence of regression, presence, and type of TILs) were differently evaluated. Twenty-two out of 27 (81.5%) Type III cases came from centers with a small caseload, while the remaining 5 (18.5%) cases came from centers with a large caseload.

**Figure 6:**
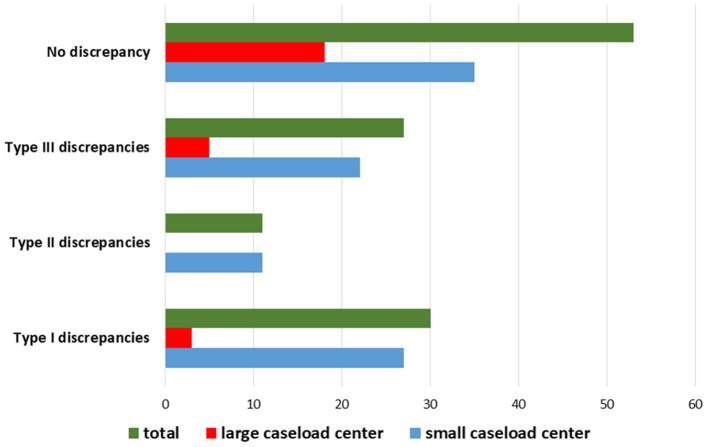
Distribution of diagnostic discrepancies according to the caseload of the hospital center in which the first diagnosis was performed.

**Figure 7:**
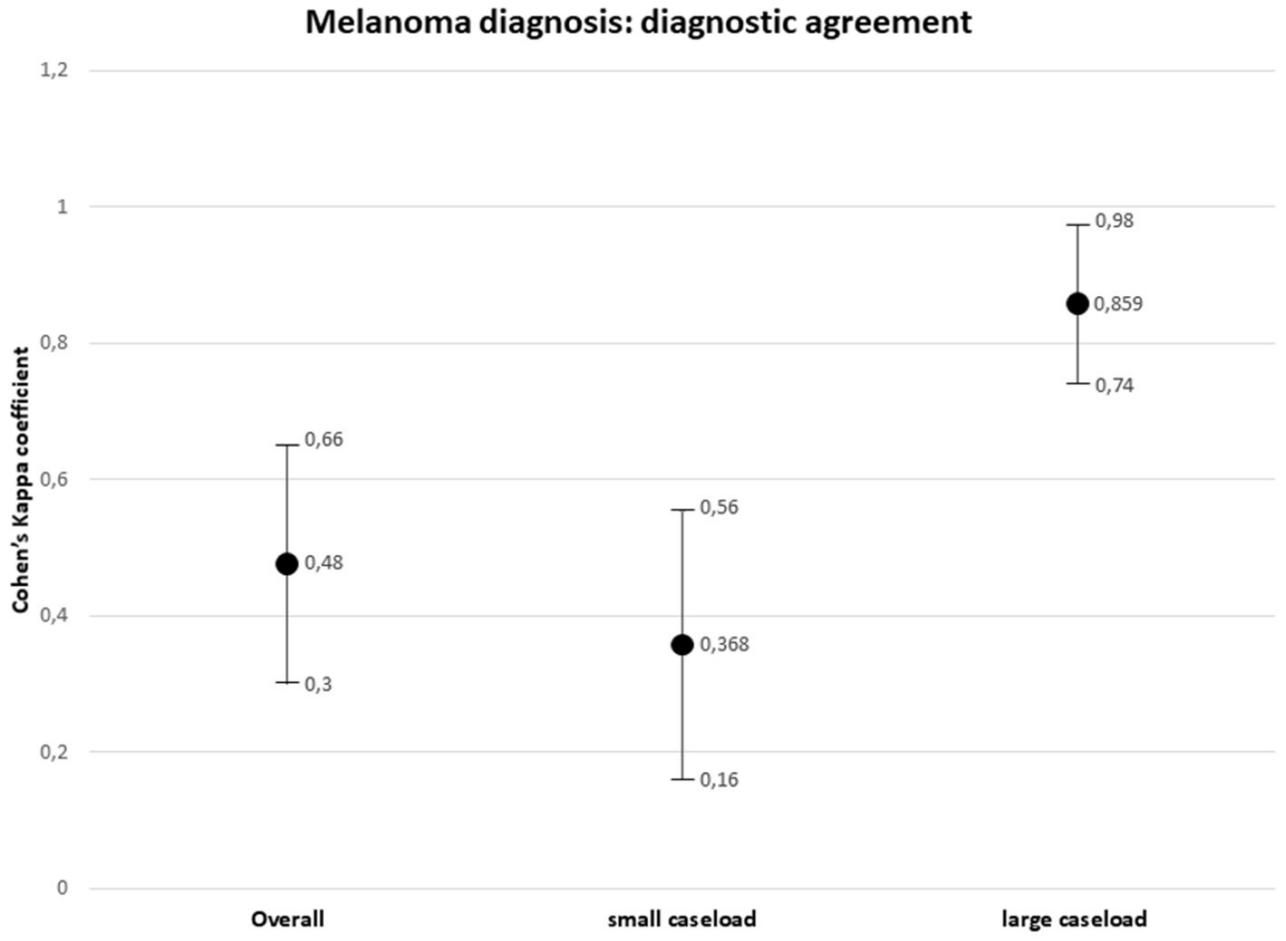
Diagnostic agreement on diagnosis of cutaneous melanoma.

The results are summarized in Table 2. The distribution of the diagnostic discrepancies according to the caseload of the hospital centers is shown in Figure 6. The diagnostic agreement values are shown in Figure 7.

**Table 2:** Diagnostic discrepancies between first diagnoses and second diagnostic opinions.

**Type of discrepancy**	***N* (%)**
No discrepancy (correspondence between the two diagnoses)	53 (44%)
Type I (opposite diagnosis)	30 (25%)
First diagnosis: MM; second diagnosis: benign nevus	10 (8.3%)
First diagnosis: borderline/indefinite lesion; second diagnosis: MM	8 (6.6%)
First diagnosis: borderline/indefinite lesion; second diagnosis: benign nevus	5 (4.1%)
First diagnosis: benign nevus; second diagnosis: MM	5 (4.1%)
First diagnosis: benign nevus; second diagnosis: borderline/indefinite lesion	1 (0.8%)
First diagnosis: benign nevus; second diagnosis: not melanocytic lesion	1 (0.8%)
Type II (affecting therapy and clinical management)	11 (9%)
Breslow thickness	9 (7.4%)
Presence of ulceration	1 (0.8%)
Breslow thickness + presence of ulceration	1 (0.8%)
Type III (affecting prognosis, but not therapy and clinical management)	27 (22%)
Breslow thickness	18 (14.9%)
Presence of ulceration	1 (0.8%)
Breslow thickness + presence of ulceration	2 (1.6%)
Other (number of mitoses, presence of regression, TILs)	6 (5%)
Total	121 (100%)

### Difference in the Clinical Management of the Patients: Clinical Impact of the SDO

**Figure 8:**
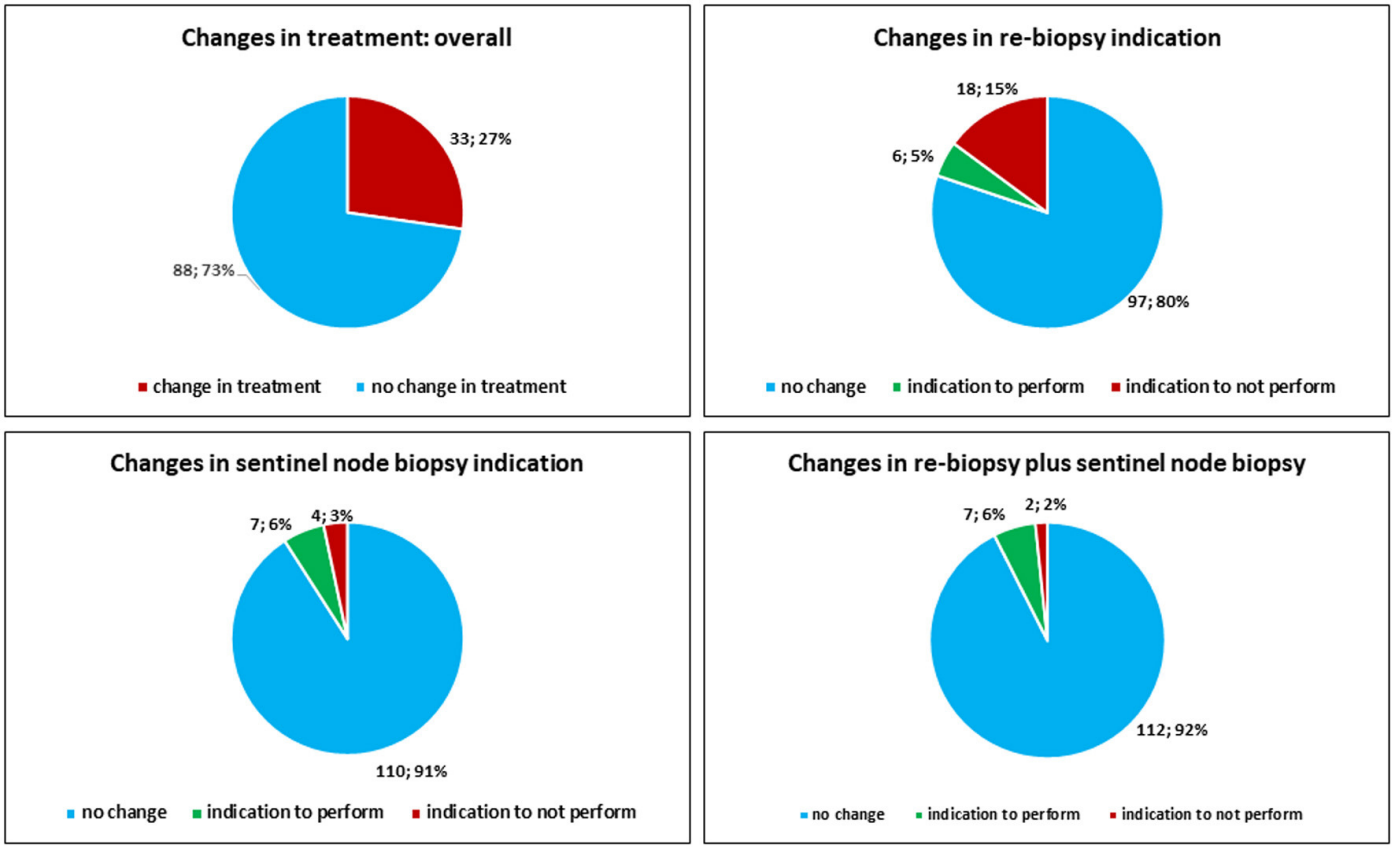
Changes in treatment indication after the SDO.

Overall, the SDO changed the clinical management (execution of re-biopsy and eventually sentinel node biopsy) of the patients in 33 out of 121 (27.3%) cases. The SDO changed the indication to perform re-biopsy alone in 24 out of 121 (19.8%) cases. In particular, the SDO removed the indication to perform re-biopsy alone in 18 out of 121 (14.8%) cases, while it posed the indication to perform re-biopsy alone in 6 out of 121 (4.9%) cases. The SDO changed the indication to perform sentinel node biopsy alone in 11 out of 121 (9.1%) cases. In particular, the SDO spurred the previously unrecognized need for sentinel-node biopsy in 7 (5.8%) cases, while in 4 (3.3%) cases the indication was removed by the SDO. The SDO removed the indication to perform re-biopsy plus sentinel node biopsy in 2 out of 121 (1.7%) cases, while it posed the indication to perform re-biopsy plus sentinel node biopsy in 7 out of 121 (5.8%) cases (Figure 8).

Concerning the cases from large caseload centers, the SDO changed the clinical management (execution of re-biopsy and eventually sentinel node biopsy) of the patients in 3 out of 26 (11.5%) cases. The clinical management (execution of re-biopsy and eventually sentinel node biopsy) was changed by SDO in 30 out of 95 (31.5%) cases from small caseload centers. The indication to execute sentinel lymph-node biopsy was changed by SDO in 11 out of 95 (11.6%) cases from small caseload centers, while there was perfect concordance between SDO and large caseload centers in indicating sentinel lymph-node biopsy.

The consistent revisions in our series significantly related to the addressing to re-biopsy (*p* = 0.000), re-biopsy plus sentinel node biopsy (*p* = 0.000), and sentinel node only (*p* = 0.000); the consistent revision was significantly related to small center origin (*p* = 0.006), but no relation with impacting clinical management was observed when considering small or large caseload centers.

The overall median time interval between the first diagnosis and the SDO resulted to be 29 days. In details, the median time intervals resulted to be 30, 22, and 28 days for type I, type II, and type III discordances, respectively. The overall mean time interval was 35 days. In detail, the mean time intervals resulted to be 34.6 days (ranging from 12 to 45 days) for type I discordances, 26.4 days (ranging from 12 to 48 days) for type II discordances, and 35 days (ranging from 8 to 46 days) for type III discordances.

## Discussion

Melanocytic lesions as one of the biggest challenges in the field of surgical pathology and the histological diagnosis of melanocytic neoplasms require a high expertise for an accurate and reproducible interpretation of morphological clues (16). In fact, the histological assessment of melanocytic neoplasms is largely based on interpretative clues, and it is strictly dependent on several non-histological findings, such as patient age and location of the lesion. Furthermore, the absence of fully specific immunohistochemical and molecular features, the rarity of some benign and malignant entities, and the remarkable overlapping between some of them contribute to generation of confusion in the diagnostic process. Interobserver concordance between different pathologists is an open issue of dermatopathology. Elmore et al. have recently interviewed 187 general pathologists from 10 US states about their confidence in diagnosing melanocytic neoplasms (16). The Authors calculated the intra-observer and interobserver concordance in the same group of pathologists when interpreting sets of cases: the intra-observer concordance resulted to be 67% and interobserver concordance resulted to be only 55% (16). These unsatisfactory diagnostic performances are responsible of detrimental clinical consequences, with some patients not receiving the most appropriate treatment. The role of the SDO in melanocytic pathology is poorly defined, and only few studies to date have focused on its impact on the clinical management of the patients. Suzuki NM et al. analyzed a Series of 31 melanocytic lesions submitted to a SDO (17). The Authors found that the final diagnosis was radically changed by the second opinion in 19% (6 out of 31) of the cases, while a therapeutic approach was changed by the second opinion in 42% (13 out of 31) of the cases (17). Gaudi et al. investigated the discrepancies between first diagnoses and SDOs in 405 cases assigned into 1 of 4 categories: melanocytic neoplasm, non-melanocytic neoplasm, inflammatory, and other. The 91 cases with major discrepancies were categorized as 36 non-melanocytic neoplasms (40%), 30 inflammatory neoplasms (33%), 23 melanocytic neoplasms (25%), and 2 other (2%) (18). Moreover, it emerged that most diagnostic discrepancies with serious clinical consequences could be attributed to pathologists lacking an explicit training in dermatopathology. Indeed, in 84% of their cases, the first diagnostic report was signed out by a pathologist devoid of a specific dermatopathology fellowship. Recently, Bhoyrul et al. (11) have evaluated the impact of a specialized SDO in a Series of 341 primary MMs. The Authors observed significant changes in the clinical management of the patients and a modification of the stage in 2.9 and in 6.7% of the cases, respectively. The results of these studies are concordant with our observations, suggesting that an SDO from an experienced dermatopathologist changes, and may improve, the clinical management of patients affected by melanocytic neoplasms. Pathologists are generally conscious about the complexities in the evaluation of melanocytic neoplasms, and, therefore, they tend to look with favor on the opportunity of a histological revision. Geller et al. (19) surveyed 207 pathologists in 10 US states to explore their feelings about SDO. The interviewed pathologists stated that they are likely to seek for a second opinion when diagnosing a borderline/intermediate neoplasm: a second opinion was requested in 85% of MELTUMPs and 88% of ASTs. Interestingly, in our Study we observed that the SDO reduced the number of lesions diagnosed as borderline/indeterminate neoplasms, recategorizing them as benign nevus or melanoma. Indeed, borderline/indeterminate neoplasms account for 15.3% of first diagnoses in our series and 4.1% of SDO. Moreover, most of the interviewed general pathologists declared their conviction that second opinion improves diagnostic accuracy (96% of the interviewed pathologists) and protects against legal problems (82% of the interviewed pathologists). As the interpretation of melanocytic neoplasms represents one of the most difficult tasks for general pathologists, diagnostic accuracy would certainly improve by getting the diagnoses verified by a specialized dermatopathologist with high expertise. In this context, the multidisciplinary oncological team may play an important role, as the convergence of different skills, including clinical examination, dermoscopy, and histology, may improve the diagnostic performance in difficult cases (11). The diagnosis of CM performed in the context of a referral Melanoma Unit with a large caseload of melanocytic lesions, embracing different professional competences and assuring clinical–pathological correlation, may improve the diagnostic accuracy with obvious benefits for the patients. Dermatologically, the evolving development of dermoscopy in expert hands allows the recognition of more and more subtle and focal atypical features in melanocytic lesions and therefore significantly improves the clinical diagnosis of CM (20). As the diagnosis of CM remains mainly morphological and interpretative, the pathologist must develop a great expertise to reach enough level of confidence in this field, gained only in centers with a large caseload of melanocytic lesions. In this context, the role of the SDO in melanocytic pathology is poorly defined and only few studies to date have focused on its impact on the clinical management of the patients. The results of this study demonstrate that SDO in the context of a multidisciplinary oncological team improves the diagnostic accuracy in selected difficult melanocytic lesions, significantly influencing the clinical choices and the management of the patients. In our series, all the clinical, dermoscopic, and histological findings were evaluated in the context of a multidisciplinary oncological team, allowing to elect the cases that need further study for a precise diagnosis. We retrospectively analyzed a series of 121 melanocytic neoplasms originally diagnosed by pathologists in small caseload centers (peripheral hospitals and private centers) and large caseload centers and then reevaluated by two experienced dermatopathologists in our reference center in the context of a multidisciplinary oncological team (Melanoma Unit). In our series, the overall agreement between the external and the referral pathologists in melanoma diagnosis resulted weak (*k* = 0.48, 95% CI, *p* = 0.000). Diagnostic discrepancies between the first diagnoses and the SDO were observed in 56% (68 out of 121) of cases. More in details, in 25% of the cases the final diagnosis was radically different, and over-interpretation and under-interpretation were observed in 50 and 46.7% of these cases, respectively. Five out of 121 (4.1%) cases originally diagnosed as benign nevi were changed to melanoma by SDO. These cases were represented by lesions arising in patients aged from 59 to 75 years in not sun-exposed locations, except one lesion located on the face. Dermoscopy was available in these cases and oriented the clinician's diagnostic opinion to atypical lesion. Histologically, these were subtle lesions with nevoid features, as some malignant features (like dermal mitoses) resulted to be evident only evaluating several levels and performing immunohistochemical tests. This series confirmed that the diagnostic agreement depends on the experience of the pathologist, as it resulted minimal between SDO and small caseload centers (*k* = 0.368, 95% CI, *p* = 0.000) and strong between SDO and large caseload centers (*k* = 0.859, 95% CI, *p* = 0.000). In addition, some features with prognostic significance (albeit with no impact on the clinical management of the patients)—like tumor-infiltrating lymphocytes, presence of regression, number of mitotic figures, etc.—were differently assessed by the SDO in 22% of the cases. These results demonstrate that the diagnosis of melanocytic lesions is strictly dependent on the experience of the pathologist and that an SDO in a referral unit may significantly improve the diagnostic accuracy. In most cases, the discrepancies between the first diagnoses and the SDOs could be simply explained by a different diagnostic interpretation of the morphological features of the lesions. However, in some cases, the observed discrepancies were linked to a technical approach, such as the examination of a larger number of histological levels from the paraffin-embedded tumoral tissue in order to detect eventual ulceration areas. For instance, an area of microscopic ulceration was detected in the case number 1 of our Series only after the examination of multiple histological levels (Figure 2).

Importantly, the SDO showed a significant clinical impact, as it changed the clinical history of the patients, influencing their therapy or their clinical management, in 27.3% of the cases. At the end, the final question we consider is if the SDO significantly changed the patients' management in our series. Thus, consistent revision significantly related with any change of treatment, i.e., re-biopsy only (*p* = 0.000), re-biopsy plus sentinel node biopsy (*p* = 0.000), and sentinel node only (*p* = 0.000). The SDO, therefore, played a crucial role to adjust the clinical management and therapy of the patients. Seven out of 121 (5.8%) patients were submitted to sentinel node biopsy thanks to the SDO (and the sentinel node resulted histologically metastatic in one case), while the indication to perform sentinel node biopsy was removed by SDO in 4 (3.3%) cases. The overall median time interval between first diagnoses and SDOs resulted to be 29 days and consequently did not compromise the chance to perform re-biopsy and sentinel biopsy within the right time (21).

As the SDO allowed to apply the best treatment to patients, we can also speculate that the SDO may play a role in reducing healthcare spending. Indeed, CM is an important cause of public health spending, with an average annual cost per patient of about $6,551 (22). In our series, the SDO allowed to avoid under-treatment in 14 cases: we can speculate that the SDO allows to save up to $91,714 per year per patient, if the patients had developed advanced melanoma in subsequent years due to under-treatment.

In conclusion, the evaluation of melanocytic neoplasms is universally recognized as one of the more difficult challenges in pathology. Consequently, the SDO performed by expert dermatopathologist, in the context of a multidisciplinary Melanoma Unit with a large caseload of melanocytic lesions, may improve the diagnostic accuracy with significant changes in the clinical management of the patients.

## Data Availability Statement

The raw data supporting the conclusions of this article will be made available by the authors, without undue reservation.

## Ethics Statement

The studies involving human participants were reviewed and approved by Ethic Commitee of Azienda Policlinico Vanvitelli, Naples; approval number 0013371/i of 06/03/2020. The patients/participants provided their written informed consent to participate in this study.

## Author Contributions

AR, FP, and RF revised the histological slides, designed the study, and contributed to the manuscript editing. GA, GB, and EM provided the clinical data and analyzed the clinical impact of second diagnostic opinion. FZ, GS, and RA performed data analysis and statistical analysis. All authors read and approved the final manuscript.

## Conflict of Interest

The authors declare that the research was conducted in the absence of any commercial or financial relationships that could be construed as a potential conflict of interest.
